# The Advisory Committee on Immunization Practices’ Ethical Principles for Allocating Initial Supplies of COVID-19 Vaccine — United States, 2020

**DOI:** 10.15585/mmwr.mm6947e3

**Published:** 2020-11-27

**Authors:** Nancy McClung, Mary Chamberland, Kathy Kinlaw, Dayna Bowen Matthew, Megan Wallace, Beth P. Bell, Grace M. Lee, H. Keipp Talbot, José R. Romero, Sara E. Oliver, Kathleen Dooling

**Affiliations:** ^1^CDC COVID-19 Response Team; ^2^General Dynamics Information Technology, Falls Church, Virginia; ^3^Emory University, Atlanta, Georgia; ^4^George Washington University Law School, Washington, D.C.; ^5^Epidemic Intelligence Service, CDC; ^6^University of Washington, Seattle, Washington; ^7^Stanford University School of Medicine, Stanford, California; ^8^Vanderbilt University School of Medicine, Nashville, Tennessee; ^9^Arkansas Department of Health.

To reduce the spread of SARS-CoV-2, the virus that causes coronavirus disease 2019 (COVID-19) and its associated impacts on health and society, COVID-19 vaccines are essential. The U.S. government is working to produce and deliver safe and effective COVID-19 vaccines for the entire U.S. population. The Advisory Committee on Immunization Practices (ACIP)* has broadly outlined its approach for developing recommendations for the use of each COVID-19 vaccine authorized or approved by the Food and Drug Administration (FDA) for Emergency Use Authorization or licensure (*1*). ACIP’s recommendation process includes an explicit and transparent evidence-based method for assessing a vaccine’s safety and efficacy as well as consideration of other factors, including implementation (*2*). Because the initial supply of vaccine will likely be limited, ACIP will also recommend which groups should receive the earliest allocations of vaccine. The ACIP COVID-19 Vaccines Work Group and consultants with expertise in ethics and health equity considered external expert committee reports and published literature and deliberated the ethical issues associated with COVID-19 vaccine allocation decisions. The purpose of this report is to describe the four ethical principles that will assist ACIP in formulating recommendations for the allocation of COVID-19 vaccine while supply is limited, in addition to scientific data and implementation feasibility: 1) maximize benefits and minimize harms; 2) promote justice; 3) mitigate health inequities; and 4) promote transparency. These principles can also aid state, tribal, local, and territorial public health authorities as they develop vaccine implementation strategies within their own communities based on ACIP recommendations.

The ACIP COVID-19 Vaccines Work Group has met several times per month (approximately 25 meetings) since its establishment in April 2020. Work Group discussions included review of the epidemiology of COVID-19 and consultation with experts in ethics and health equity to inform the development of an ethically principled decision-making process. The Work Group reviewed the relevant literature, including frameworks for pandemic influenza planning and COVID-19 vaccine allocation (*3*–*8*); summarized this information; and presented it to ACIP. ACIP supported four fundamental ethical principles to guide COVID-19 vaccine allocation decisions in the setting of a constrained supply. Essential questions that derive from these principles can assist in vaccine allocation planning (Table 1).

**TABLE 1:** Essential questions for COVID-19 vaccine allocation planning related to ethical principles — United States, 2020

Ethical principle	Essential question
**Maximize benefits and minimize harms**	What groups are at highest risk for SARS-CoV-2 infection, COVID-19 disease, hospitalization, and death?
	What groups are essential to the COVID-19 response?
	What groups are essential to maintaining critical functions of society?
	What are the important characteristics of these groups (e.g., size or geographic distribution) that might inform the magnitude of benefit based on the amount of vaccine available or its characteristics?
**Promote justice**	Does the allocation plan result in fair and equitable access of the vaccine for all groups?
	How do characteristics of the vaccine and logistical considerations affect fair access for all persons?
	Does allocation planning include input from groups who are disproportionately affected by COVID-19 or face health inequities resulting from social determinants of health, such as income and health care access?
**Mitigate health inequities**	Does the plan identify and address barriers to vaccination among any groups who are disproportionately affected by COVID-19 or who face health inequities resulting from social determinants of health, such as income and health care access?
	Does the allocation plan contribute to a reduction in health disparities in COVID-19 disease and death?
	What health inequities might inadvertently result from the allocation plan, and what interventions could remove or reduce them?
	Is there a mechanism for timely assessment of vaccination coverage among groups experiencing disadvantage and the possibility for course correction if inequities are identified?
**Promote transparency**	How does development of the allocation plan include diverse input, and if possible, public engagement?
	Are the allocation plan and evidence-based methods publicly available?
	Is the allocation plan clear about what is known and unknown and about the quality of available evidence?
	What is the process for revision of allocation plans based on new information?
	Is there a mechanism to report demographic data elements for vaccine recipients (e.g., age, race/ethnicity, and occupation) to support equitable vaccination coverage?

**Abbreviation:** COVID-19 = coronavirus disease 2019.

**Maximize benefits and minimize harms.** Allocation of COVID-19 vaccine should maximize the benefits of vaccination to both individual recipients and the population overall. These benefits include the reduction of SARS-CoV-2 infections and COVID-19–associated morbidity and mortality, which in turn reduces the burden on strained health care capacity and facilities; preservation of services essential to the COVID-19 response; and maintenance of overall societal functioning. Identification of groups whose receipt of the vaccine would lead to the greatest benefit should be based on scientific evidence, accounting for those at highest risk for SARS-CoV-2 infection or severe COVID-19–related disease or death, and the essential role of certain workers. The ability of essential workers, including health care workers and non–health care workers, to remain healthy has a multiplier effect (i.e., their ability to remain healthy helps to protect the health of others or to minimize societal and economic disruption). Some of these workers are at increased risk for SARS-CoV-2 infection because of their limited ability to maintain physical distance in the workplace or because they do not have consistent access to recommended personal protective equipment.

**Promote justice.** Inherent in the principle of justice is an obligation to protect and advance equal opportunity for all persons to enjoy the maximal health and well-being possible. Justice rests on the belief in the fundamental value and dignity of all persons. Allocation of COVID-19 vaccine should promote justice by intentionally ensuring that all persons have equal opportunity to be vaccinated, both within the groups recommended for initial vaccination, and as vaccine becomes more widely available. This includes a commitment to removing unfair, unjust, and avoidable barriers to vaccination that disproportionately affect groups that have been economically or socially marginalized, as well as a fair and consistent implementation process. Input from a range of external entities, partners, and community representatives is particularly important in developing and assessing allocation plans.

**Mitigate health inequities.** Health equity is achieved when every person has the opportunity to attain his or her full health potential and no one is disadvantaged from achieving this potential because of social position or other socially determined circumstances.^†^ Disparities in the severity of COVID-19 and COVID-19–related death, as well as inequities in social determinants of health that are linked to COVID-19 risk, such as income or health care access and utilization, are well documented among certain racial and ethnic minority groups (*9*). Vaccine allocation strategies should aim to both reduce existing disparities and to not create new disparities. Efforts should be made to identify and remove obstacles and barriers to receiving COVID-19 vaccine, including limited access to health care or residence in rural, hard-to-reach areas.

**Promote transparency.** Transparency relates to the decision-making process and is essential to building and maintaining public trust during vaccine program planning and implementation. The underlying principles, decision-making processes, and plans for COVID-19 vaccine allocation must be evidence-based, clear, understandable, and publicly available. To the extent possible, considering the urgency of the COVID-19 response, public participation in the creation and review of the decision-making process should be facilitated. In addition, when feasible, tracking administration of vaccine to the groups recommended for initial vaccine allocation can contribute to transparency and trust in the process. In an ongoing public health response, the situation continually evolves as new information becomes available. Transparency includes being clear about the level of certainty in the available evidence and communicating new information that might change recommendations in a timely fashion.

For the period when the supply of COVID-19 vaccine will be limited, ACIP has considered four groups for initial vaccine allocation. These include health care personnel, other essential workers, adults with high-risk medical conditions, and adults aged ≥65 years (including residents of long-term care facilities) (Table 2). These groups were selected based on available scientific data, vaccine implementation considerations, and ethical principles. The principle of transparency is applied across the entirety of the vaccine allocation decision-making process. ACIP’s meetings are open to the public, meeting minutes and archived webcasts are available online, and data (including data from vaccine clinical trials) and analytic methods used in developing ACIP recommendations are publicly available.^§^ Members of the public are invited to submit written comments to the Federal Register or provide oral comment during ACIP meetings. ACIP’s 30 nonvoting representatives from liaison organizations facilitate engagement with professional medical and public health organizations and other stakeholders and partners.

**TABLE 2:** Application of ethical principles to four candidate groups for initial COVID-19 vaccine allocation — United States, 2020

Principles (with transparency across the decision-making process)	Candidate groups* (approximate no.)			
	Health care personnel^†^ (21 million)	Other essential workers^†^ (87 million)	Adults with high-risk medical conditions^§^ (>100 million)	Adults aged ≥65 years (53 million)
**Maximize benefits and minimize harms**	Preserves health care services essential to the COVID-19 response and the overall health care system	Preserves services essential to the COVID-19 response and overall functioning of society	Reduces morbidity and mortality in persons with high incidence of COVID-19 disease and death**	Reduces morbidity and mortality in persons with high incidence of COVID-19 disease and death^††^
	Multiplier effect^¶^	Multiplier effect^¶^		
**Promote justice**	Addresses elevated occupational risk for SARS-CoV-2 exposure for those unable to work from home	Addresses elevated occupational risk for SARS-CoV-2 exposure for those unable to work from home	Will require focused outreach to vaccinate persons in this group who have no or limited access to health care or experience inequities in social determinants of health	Will require focused outreach to vaccinate persons in this group who have no or limited access to health care or experience inequities in social determinants of health
	Promotes access to vaccine across a spectrum of HCP job types and settings	Promotes access to vaccine and reduces barriers to vaccination in occupations with low vaccine uptake^§§^		
**Mitigate health inequities**	Racial and ethnic minority groups are disproportionately represented in low-wage HCP^¶¶^	Racial and ethnic minority groups are disproportionately represented in many essential industries***	Increased prevalence of obesity and diabetes (most prevalent conditions in this group) among some racial and ethnic minority groups; increased prevalence of some medical conditions for persons in rural areas^§§§^	Although racial and ethnic minority groups are underrepresented among adults aged ≥65 years, certain groups have disproportionate COVID-19–related hospitalization and death rates^¶¶¶^
		Approximately one quarter of essential workers live in low-income families^†††^	Could increase health inequities because diagnosis of high-risk medical conditions requires access to health care	Strict age-based criterion could increase disparities due to racial and social inequities, such as occupation, income, access to health care

**Abbreviations:** COVID-19 = coronavirus disease 2019; HCP = health care personnel.

* Health care personnel: paid and unpaid persons serving in health care settings who have the potential for direct or indirect exposure to patients or infectious materials; other essential workers: person who conduct operations vital for continuing critical infrastructure, such as food, agriculture, transportation, education, and law enforcement; adults with high risk medical conditions: adults who have one or more high-risk medical conditions, such as obesity, diabetes, and cardiovascular disease; adults aged ≥65 years: includes adults living at home and approximately 3 million living in long-term care facilities. There is considerable overlap between groups, for example, many adults aged ≥65 years also have high-risk medical conditions.

^†^ Essential workers during the COVID-19 response have been defined by the U.S. Department of Homeland Security Cybersecurity and Infrastructure Security Agency. https://www.cisa.gov/sites/default/files/publications/Version_4.0_CISA_Guidance_on_Essential_Critical_Infrastructure_Workers_FINAL%20AUG%2018v2_0.pdf.

^§^ Medical conditions considered high-risk are updated routinely based on the best available scientific data: https://www.cdc.gov/coronavirus/2019-ncov/need-extra-precautions/people-with-medical-conditions.html.

^¶^ The ability of one or more groups to remain healthy helps protect the health of others and/or minimize disruption to society and the economy.

** As of October 31, 2020, nearly 90% of persons with COVID-19–associated hospitalizations have at least one high-risk condition. Data are routinely updated through COVID-19–Associated Hospitalization Surveillance Network (COVID-NET) (https://gis.cdc.gov/grasp/COVIDNet/COVID19_5.html); in-hospital deaths reported to COVID-NET during March–May, 2020 were associated with certain underlying medical conditions (https://academic.oup.com/cid/advance-article/doi/10.1093/cid/ciaa1012/5872581).

^††^ As of November 12, 2020, 80% of COVID-19 deaths were among adults aged ≥65 years. Data are routinely updated through CDC case-based surveillance (https://covid.cdc.gov/covid-data-tracker/#demographics); long-term care residents account for a large proportion of deaths among adults aged ≥65 years (https://data.cms.gov/stories/s/COVID-19-Nursing-Home-Data/bkwz-xpvg/).

^§§^ Influenza vaccination coverage is low among many non–health care essential workers; such coverage is lowest among construction workers (10.7%) (https://www.cdc.gov/niosh/docs/2012-161/pdfs/2012-161.pdf?id = 10.26616/NIOSHPUB2012161).

^¶¶^ Health Resources and Services Administration estimates from American Community Survey 2011–2015 (https://bhw.hrsa.gov/sites/default/files/bhw/nchwa/diversityushealthoccupationstechnical.pdf).

*** Among 742 food and agriculture workplaces in 30 states, 73% of workers were Hispanic or Latino and 83% of COVID-19 cases occurred in racial or ethnic minority workers (https://wwwnc.cdc.gov/eid/article/27/1/20-3821_article).

^†††^ Center for Economic and Policy Research estimates from American Community Survey, 2014–2018 (https://cepr.net/a-basic-demographic-profile-of-workers-in-frontline-industries).

^§§§^ National Center for Health Statistics. National Health Interview Survey, 2018. Estimates not available for Hawaiian/other Pacific Islander persons or for chronic kidney disease among American Indian/Alaska Native persons (https://www.cdc.gov/nchs/nhis/ADULTS/www/index.htm; https://www.cdc.gov/mmwr/volumes/69/wr/mm6929a1.htm).

^¶¶¶^ As of October 31, 2020, compared with COVID-19 hospitalization rates for adults aged ≥65 years who are non-Hispanic White, such rates were higher among adults aged ≥65 years who were non-Hispanic Black (rate ratio [RR] = 3.3), Hispanic or Latino (RR = 2.6), and non-Hispanic American Indian or Alaska Native (RR = 2.4). Data are routinely updated through COVID-NET (https://www.cdc.gov/coronavirus/2019-ncov/covid-data/covidview/index.html); adults aged ≥65 years who are Hispanic or non-Hispanic Black experience disproportionate COVID-19–associated death rates (https://www.cdc.gov/nchs/nvss/vsrr/covid19/health_disparities.htm).

All four groups proposed for initial allocation of COVID-19 vaccine merit strong consideration from an ethical perspective. Current planning scenarios estimate, however, that the expected number of doses during the first weeks of vaccine distribution might only be sufficient to vaccinate approximately 20 million persons.^¶^ Although there is considerable overlap between groups** (*10*), the initial supply will not be adequate to vaccinate the entirety of all four groups; for example, there are approximately 100 million health care personnel and essential workers (Table 2). Published frameworks for COVID-19 allocation and ACIP discussions indicate a clear consensus that the first allocation of COVID-19 vaccine supplies should be directed to health care personnel (*1*,*5*–*8*); discussion of allocation to the other three groups is ongoing. As additional vaccine supplies become available, other groups may be vaccinated concurrent with health care personnel.

## Discussion

During a pandemic, ethical guidelines can help steer and support decisions around prioritization of limited resources (*3*,*4*). Consideration of ethical values and principles has featured prominently in discussions about allocation of COVID-19 vaccines. This consideration is particularly relevant because the COVID-19 pandemic has highlighted long-standing, systemic health and social inequities. Although various frameworks for COVID-19 vaccine allocation demonstrate differences in their structure (e.g., based on varying combinations of different goals, objectives, criteria, and other structural elements) and emphasis (e.g., inclusion of global and national considerations), nearly all reference values and principles similar to those which ACIP considers fundamental (*5*–*8*). ACIP viewed the following characteristics as critical for its ethical approach to COVID-19 vaccine allocation when supply is limited: simplicity in structure and definitions; acceptability to stakeholders; and ease of application, both at the national and state, tribal, local, and territorial levels.

Allocation of limited vaccine supplies is complicated by efforts to address the multiple goals of a vaccine program, most notably those related to the reduction of morbidity and mortality and the minimization of disruption to society and the economy. If the goals of a pandemic vaccination program are not clearly articulated and prioritized, drawing distinctions between groups that merit consideration for allocation of vaccine when supply is constrained can become difficult. The unanimity in opinion for early vaccination of health care personnel indicates that maintenance of health care capacity has emerged as a high priority in the context of a severe pandemic. This perspective aligns with ethical considerations for pandemic influenza planning (*3*,*4*). If vaccine supply remains constrained, it might be necessary to identify subsets of other groups for subsequent early allocation of COVID-19 vaccine. At the national, state, tribal, local, and territorial levels, such decisions should be guided, in part, by ethical principles and consideration of essential questions, with particular consideration of mitigation of health inequities in persons experiencing disproportionate COVID-19 morbidity and mortality. In the setting of a constrained supply, the benefits of vaccination will be delayed for some persons; however, as supply increases, there will eventually be enough vaccine for everyone.

In addition to ethical considerations, ACIP’s recommendations regarding receipt of the initial allocations of COVID-19 vaccine during the period of constrained supply will be based on science (e.g., available information about the vaccine’s characteristics such as safety and efficacy in older adults and epidemiologic risk) and feasibility of implementation (e.g., storage and handling requirements). Thus, ACIP’s allocation recommendations will be made in conjunction with specific recommendations for the use of each FDA-authorized or licensed COVID-19 vaccine. Although the ethical principles in this report are fundamental for stewardship of limited vaccine supply, they can also be applied when COVID-19 vaccines are widely available, to ensure equitable and just access for all persons.

**Summary**

**What is already known about this topic?**

During the period when the U.S. supply of COVID-19 vaccines is limited, the Advisory Committee on Immunization Practices (ACIP) will make vaccine allocation recommendations.

**What is added by this report?**

In addition to scientific data and implementation feasibility, four ethical principles will assist ACIP in formulating recommendations for the initial allocation of COVID-19 vaccine: 1) maximizing benefits and minimizing harms; 2) promoting justice; 3) mitigating health inequities; and 4) promoting transparency.

**What are the implications for public health practice?**

Ethical principles will aid ACIP in making vaccine allocation recommendations and state, tribal, local, and territorial public health authorities in developing vaccine implementation strategies based on ACIP’s recommendations.

## Footnotes

* The ACIP includes 15 voting members responsible for making vaccine recommendations. Fourteen of the members have expertise in vaccinology, immunology, pediatrics, internal medicine, nursing, family medicine, virology, public health, infectious diseases, and/or preventive medicine; one member is a consumer representative who provides perspectives on the social and community aspects of vaccination. In addition to the 15 voting members, ACIP includes eight ex officio members who represent other federal agencies with responsibility for immunization programs in the United States, and 30 nonvoting representatives of liaison organizations that bring related immunization expertise. https://www.cdc.gov/vaccines/acip/members/index.html.

† https://www.cdc.gov/chronicdisease/healthequity/index.htm.

§ https://www.cdc.gov/vaccines/acip/index.html.

¶
https://www.cdc.gov/vaccines/imz-managers/downloads/COVID-19-Vaccination-Program-Interim_Playbook.pdf .

** There is overlap among these four groups. For example, in one analysis, among the 3.8% of U.S. adults who work directly with patients as health care workers, 38.6% have high-risk medical conditions or are aged >65 years.
